# Preteen Suicidal Ideation and Adolescent Academic Well-Being Among Child Welfare-involved Youth

**DOI:** 10.1007/s12310-024-09726-x

**Published:** 2024-11-22

**Authors:** Nathaniel W. Anderson, Gabriel W. Hassler, Elie Ohana, Beth Ann Griffin, Arielle H. Sheftall, Lynsay Ayer

**Affiliations:** 1 Department of Health Policy and Management, University of California Los Angeles, Los Angeles, USA; 2 RAND Corporation, 1776 Main St, Santa Monica, CA 90401 USA; 3 RAND Corporation, 1200 South Hayes St, Arlington, VA 22202 USA; 4 Department of Psychiatry, University of Rochester Medical Center, Rochester, USA

**Keywords:** Suicidal ideation, School, Well-being, Adolescent health

## Abstract

**Background:**

Youth involved in the U.S. child welfare system (CWS) are at risk for mental health problems, including suicidal ideation (SI). However, the relationship between preteen suicidal ideation and academic outcomes has not been considered.

**Methods:**

This study uses data from two nationally representative longitudinal surveys of CWS-involved youth to examine the association between preteen suicidal ideation (ages 7–11) and subsequent academic well-being (ages 12–17) among CWS-involved youth in the United States. Suicidal ideation was assessed using a single self-report item. Academic well-being was assessed through a number of constructs related to young people’s ability to thrive in the present and future, including school engagement, academic achievement, and expectations of what their lives would look like in adulthood. Linear regression models with person-level random effects were estimated.

**Results:**

Findings indicate CWS-involved youth with a history of preteen suicidal ideation performed worse across all measures of adolescent academic well-being compared to their peers without a history of suicidal ideation.

**Conclusions:**

These findings, though associational, have potentially broad implications for understanding how early life suicidal ideation may impede CWS-involved youths’ ability to thrive academically.

## Introduction

Youth involved in the U.S. child welfare system (CWS) are at especially high risk for mental health problems, including suicidal ideation (SI) (Evans et al., 2017). By some estimates, roughly a quarter of CWS-involved youth report SI, compared to approximately one-in-ten among the general population (Anderson, 2011; Evans et al., 2017). Amidst a backdrop of increases in SI and other suicide-related outcomes in young people (Bridge et al., 2023; Ruch et al., 2019; Xiao et al., 2021), a continued focus on SI among CWS-involved youth is essential for public health strategies emphasizing health equity (Braveman, 2014; Harris et al., 2020).

Researchers have attributed the higher risk for SI among CWS-involved youth to several factors. First, their experiences of maltreatment, including higher rates of physical and sexual abuse, directly increase their risk for adverse mental health outcomes, including SI and other suicide-related outcomes (Dunn et al., 2013; Miller et al., 2013). The timing of the exposure to maltreatment may also influence risk of suicidal ideation. For instance, findings from a nationally representative longitudinal study of youth found that participants first exposed to sexual abuse during early childhood had a 146% increase in the odds of suicidal ideation compared to respondents maltreated as adolescents (Dunn et al., 2013). Second, CWS-involved youth experience other adversities such as disruption of social and familial relationships that can elevate their risk of SI and other suicide-related outcomes (Conn et al., 2015; Sheftall et al., 2016). For instance, a recent retrospective matched case-control study found that CWS-involved youth that experienced out-of-home placements were twice as likely to die by suicide (Ruch et al., 2021).

### Suicidal Ideation Amongst Preteens

Research examining SI and other suicide-related outcomes in young people tends to focus on adolescence, but increasingly there is a need to expand the scope to include preteens (Ayer et al., 2020). Several population-based analyses have documented worrying increases in SI and related outcomes among the younger child population, particularly among children of minoritized racial/ethnic backgrounds (Ayer et al., 2020; Bridge et al., 2023; Liu et al., 2022). Furthermore, risk factors appear to be different for SI experienced in preteen years relative to when it occurs in adolescence. For instance, preteens with SI are more likely to be boys and more likely to have experienced attention deficit/hyperactivity disorder (ADHD) symptoms, while adolescents with SI are more likely to be girls and more likely to exhibit depressive symptoms (Adrian et al., 2016; Geoffroy et al., 2022). Lastly, the impacts of SI at earlier stages of development have particularly important implications for future health and flourishing. Lifecourse theory suggests that acute mental distress experienced in earlier phases of life can have severe consequences on development and thriving (Halfon & Hochstein, 2002; Halfon et al., 2014). Thus, documenting adverse long-term impacts of earlier exposure to SI is a critical component for building support for future programs and policy that seek to address this issue.

### Suicidal Ideation and Academic Well-Being

Academic well-being, which we define as a set of education- and achievement-related measures corresponding to how young people “experience and evaluate their lives, as well as having skills and opportunities to construct meaningful futures” (Robert Wood Johnson Foundation, 2019), is one such mechanism by which early life SI may impact future health and flourishing. Previous research has demonstrated associations between SI in youth and lower levels of school engagement, academic performance, and achievement-oriented future expectations (Carter et al., 2007; Chen et al., 2022; De Luca & Wyman, 2012; De Luca et al., 2016; Marraccini & Brier, 2017; Posick and Zimmerman, 2022; Zhu et al., 2019). However, the directionality of the relationship in these studies tends to place SI as the outcome to be studied, with measures of academic well-being assumed to be moderating or mediating factors. This overlooks evidence suggesting a bidirectional relationship between mental health and academic well-being, particularly when considering interactions between the two over time (Racine et al., 2019).

Thus the potential for SI to impact academic well-being is worrisome, particularly since constructs like school engagement, academic performance, and achievement-oriented future expectations are highly predictive of positive outcomes in adulthood, including higher educational attainment (Abbott-Chapman et al., 2014; Fraysier et al., 2020; Symonds et al., 2022), increased healthy behaviors (Brumley et al., 2017; McDade et al., 2011; Prince et al., 2019; Sipsma et al., 2015), and improved physical and mental health (Chen et al., 2023; Kim & Kim, 2020). This is a particularly salient issue for CWS-involved preteens, who tend to fare worse across each of these outcomes (Bruskas, 2008; Pears et al., 2013; Zetlin & Weinberg, 2004).

### The Current Study

This observational study addresses this gap in the literature using data from a longitudinal study of CWS-involved youth. Our aim was to investigate how children experiencing SI in their preteen years (ages 7–11) performed on several measures of academic well-being assessed during adolescence (ages 12–17) relative to their peers who did not experience SI. We hypothesized that preteens who experienced SI would subsequently be less engaged in school, perform worse on tests assessing their skills in reading and mathematics, and have reduced expectations for their future.

## Methods

### Participants

We used data from the Restricted Release versions of the National Survey of Child and Adolescent Wellbeing I and II (NSCAW I and II), two nationally representative longitudinal studies that examined the experiences of young people involved in the CWS between 1999–2007 and 2008–2012, respectively (RTI International, 2008; RTI International, 2014). The multiple waves and richness of the measures collected in these studies allow for the assessment of a broad set of outcomes related to academic well-being, making the NSCAW studies well-suited for studying mental health within the CWS population. Since both studies adopted similar sampling and measures across waves, we combined them to increase statistical power.

The NSCAW-I dataset includes 5,501 children ages birth to 14 and their families who were investigated for child abuse or neglect between October 1999 and December 2000 by child protective services (Dowd et al., 2008). Children were sampled first from 92 primary sampling units (PSUs) in 97 counties nationwide, followed by stratified random selection of children from closed investigations within those PSUs. Infants, sexual abuse cases, and cases receiving ongoing CWS services after investigation were oversampled. Face-to-face interviews with children, caregivers, and child welfare caseworkers were conducted within 2–6 months of close of the investigation (baseline), with three follow-up time points: 18 months (Wave 3; *N* = 4,470), 36 months (Wave 4; *N* = 4,511), and 59-96 months (Wave 5; *N* = 4,134).

The NSCAW-II study included 5,872 children aged birth to 17.5 years who had contact with the CWS from 2008 to 2009 and whose investigations were closed during this period (Dowd

et al., 2013). Children, caregivers, and caseworkers were interviewed face-to-face at three time points: baseline (March 2008-September 2009), and approximately 18 (Wave 2; *N* = 5,872) and 36 (Wave 3; *N* = 5,251) months following baseline. Children in NSCAW-II were selected from 81 of the original 92 NSCAW PSUs in 83 counties nationwide. Infants, children in out-of-home placements, and cases receiving services were oversampled. We excluded 727 individuals taken from a long-term foster care sample, since this component was not present in NSCAW-I.

Our analytic sample was comprised of respondents ages 7-11 at baseline who completed a follow-up wave during ages 12-17. Among NSCAW-I and NSCAW-II respondents, 41.5% and 13.0% met these criteria, respectively, which resulted in a final analytic sample size of 2,002 respondents across the two studies. Since respondents could answer multiple times during ages 12–17, the dataset consisted of 3,048 observations, or approximately 1.5 observations during adolescence per respondent. Missingness in the covariates and outcomes (ranging from 0.0% to 14.7% with mean of 4.7% in Wave 1), affected 1,796 (58.9%) of the observations. Therefore, we employed multiple imputation chained equation methods using the MICE package in R Statistical software with 100 iterations to retain the full sample.

Both NSCAW I and NSCAW II include a set of analytic weights to allow for nationally representative estimates for the CWS child population. In the interest of generalizability, we adjusted the individual-level weights from the NSCAW I dataset so that the weighted NSCAW I population statistics matched those from NSCAW II on race/ethnicity, sex at birth, age distribution, and type of alleged maltreatment, using the raking procedure described in DeBell and Krosnick (2009) (DeBell & Krosnick, 2009).

### Measures

#### Suicidal Ideation

To assess SI we used a single self-reported item from the Childhood Depression Inventory (CDI), a 27-item scale that rates the severity of symptoms related to depression in children and adolescents (Kovacs, 1992). Prior work has shown strong reliability and validity across various samples of young people (Saylor et al., 1984; Smucker et al., 1986).

The CDI SI item asks about the past 2 weeks: 1 = “I do not think about killing myself”; 2 = “I think about killing myself but I would not do it”; and 3 = “I want to kill myself.” We dichotomized the measure such that values of 2 or 3 were counted as experiencing SI, as has been done in several other studies (Anderson, 2011; Fulginiti et al., 2018). If a child endorsed SI on this item at any wave between ages 7–11, they were coded as 1 = suicidal ideation. Otherwise, they were coded as 0 = no ideation.

#### Academic Well-Being

For our outcomes, we focused on a broad set of measures related to young people’s present academic experiences, as well as their anticipated future achievement. These were assessed during ages 12–17:

**School engagement**. Respondents were asked to rate their responses to 11 questions about their school experiences based on a set of outcome measures developed for the Drug Free Schools and Communities Act passed in the 1990s (Tashjian et al., 1996). Although results from any analyses of validity and reliability were not published from the original study for which it was developed, prior analyses using NSCAW data have demonstrated its validity within this sample and a number of other studies have used aggregated the 11 items into a single composite measure for analysis (Cage et al., 2019; Cage et al., 2020). The questions asked children to evaluate on a 4-point scale (1 = “never”, 2 = “sometimes”, 3 = “often”, and 4 = “almost always”) how often they: enjoy school, hate school, try to do their best in school, find schoolwork too hard, find classes interesting, fail to complete assignments, are sent to the office, get along with their teachers, listen carefully, finish their homework, and get along with their peers. Four items are reverse-coded such that higher scores indicate greater engagement. We used the average score from this measure (α = .80), ranging from 1 to 4.

**Academic achievement*.*** We used the standard scores for Letter-Word Identification (76 items) and Applied Problems (63 items) from the Woodcock-Johnson IV (WJ IV) test, which was administered in the fifth wave of NSCAW I and in all waves of NSCAW II. The WJ IV is a reliable and valid interviewer-administered test of cognitive abilities and achievement among youth and adults (Abu-Hamour et al., 2012). Higher scores on these Letter-Word Identification and Applied Problems scales are positively associated with subsequent educational attainment (Davis-Kean et al., 2022; Jones et al., 2015).

**Future expectations*.*** Youth were asked to answer five questions about their future which were adapted from the National Longitudinal Study of Adolescent to Adult Health (Bearman et al., 1997). Specifically, youth were asked to rate their chances of living to age 35, to be married by age 25, to graduate high school, to have a good job by age 30, and to have a child and raise a family. The questions were rated on a 5-point scale (1 = “no chance”, 2 = “some chance”, 3 = “about 50–50”, 4 = “pretty likely”, and 5 = “it will happen”). In rare cases where respondents indicated they had already accomplished these (i.e., graduated high school or had children), those scores were recoded to “it will happen.” Since Cronbach’s alpha within the study sample was moderate (α = .66), we conducted a factor analysis (Appendix A), which showed that three of the items (chances to live to 35, chances to graduate high school, and chances to have a good job) formed a suitable summary measure, coded as an average ranging from 1 to 5.

#### Covariates

We selected several covariates to include in our models as potential confounders of the associations between SI and academic well-being. They include the following:

**Child demographics*.*** Child age (continuous), race/ethnicity (White, non-Hispanic; Black, non-Hispanic; Hispanic; other), and sex (male, female) were assessed by child and caregiver interviews at Wave 1. When there were discrepancies between reporters, the NSCAW I and II used an algorithm to determine the final demographic codes in a consistent way (RTI International, 2008; RTI International, 2014). We note that there was not a measure of gender identity, or a distinction made between sex at birth and gender identity.

**Child maltreatment*.*** At Wave 1, caseworkers were asked to indicate which maltreatment type, of all reported types of abuse or neglect, was “the most serious.” Response options included: physical abuse, sexual abuse, emotional abuse, physical neglect (failure to provide), neglect (lack of supervision), abandonment, moral/legal maltreatment, educational maltreatment, exploitation (e.g., sale of minor’s time or behavior), and other.

**Out-of-home placement*.*** The number of days spent in out-of-home (OOH) placements (e.g., foster care, group home) across all waves was assessed via caseworker interview. If a child had no OOH placements, then the number of days of OOH placement was zero. Thus, this quantity applies both to children placed and not placed in OOH settings.

**Baseline mental health*.*** The child behavior checklist (CBCL) (Achenbach & Rescorla, 2001) is a caregiver-reported measure of children’s psychopathology. Caregivers rated each item on a 3-point scale (0 = “not true”, 1 = “somewhat or sometimes true”, and 2 = “very true or often true”) based on the prior six months. The internalizing broadband scale is a measure of internalizing problems such as anxiety and depression, containing 33 items. The externalizing scale includes 34 items which relate to children’s externalizing behaviors such as aggression, rule breaking and hyperactivity. In this study we used the raw internalizing (Cronbach’s a = .89) and externalizing (Cronbach’s a = .88) scale scores as measures of child mental health from baseline (Wave 1).

### Procedure & Data Analysis

Since the study design is a repeated measures analysis, we used a linear regression specification with random effects for individual respondents where the outcome was the academic well-being variable at each wave. We standardized the outcome measures to assist with interpretability. All analyses were performed in STATA statistical software (Version 17.0). We followed the Strengthening the Reporting of Observational Studies in Epidemiology (STROBE) reporting guideline (Von Elm et al., 2014).

All regression models controlled for several covariates from the Wave 1 survey, when children were between 7 and 11 years old, which are potential confounders for the relationship between preteen SI and later (adolescent) academic well-being. Specifically, we included age, sex, an age-by-sex interaction, race/ethnicity, most severe form of maltreatment, existing harm from maltreatment as assessed on a 4-point scale by the caseworker, cumulative number of days in out of home placement across the entire study, internalizing and externalizing problems, and whether the respondent was sampled in NSCAW I or II. Although we report standard *p* values using an a = .05 threshold, we also assessed for multiple testing issues using a Benjamini-Hochberg correction and found reported associations were robust.

## Results

### Description of Preteens With and Without SI

Table 1 presents bivariate statistics for the variables included in the analysis, stratified by whether or not the participant experienced SI during their preteen years. In terms of observed covariates, youth experiencing SI during their preteen years were largely similar to their counterparts who did not have SI. However, youth with preteen SI were typically younger at baseline (8.8 years on average) relative to their counterparts without preteen SI (9.5 years on average [*p* value < .01]). Additionally, compared to preteens without SI, those who reported SI in their preteen years were more likely to be male (53.3% compared to 48.2% [*p* value = .04]), spend more time in out of home placements (304.8 days compared to 252.3 days [*p* value = .04]), have higher levels of internalizing (average score of 10.6 compared to 9.2 [*p* value < .01]) and externalizing (average score of 15.9 compared to 13.7 [*p* value < .01]) problems, and have higher representation in NSCAW-I (78.7% compared to 68.0% [*p* value < .01]).

**Table 1:** Descriptive statistics of analytic sample. Source: NSCAW I and II

	Suicidal ideation in preteen years (n=843)		No suicidal ideation in preteen years (n=1,159)	
Measure	Mean	Std. dev.	Mean	Std. dev.
Age at wave 1 (Years)	8.79	1.29	9.52***	1.31
*Sex (%)*				
Female	46.7%	–	51.8%**	–
Male	53.3%	–	48.2%**	–
*Race/Ethnicity (%)*				
White, non-Hispanic	41.6%	–	43.5%	–
Black, non-Hispanic	30.1%	–	30.8%	–
Hispanic	19.8%	–	18.4%	–
Other, non-Hispanic	8.5%	–	7.4%	–
*Form of abuse (%)*				
Physical maltreatment	23.2%	–	24.9%	–
Sexual maltreatment	15.9%	–	14.9%	–
Failure to supervise	25.4%	–	22.6%	–
Failure to provide	14.4%	–	12.9%	–
Other	21.0%	–	24.7%*	–
Harm from previous maltreatment (ranges from 1 to 4)	2.35	1.02	2.33	1.03
Cumulative days of out of home placements (Days)	305.28	581.00	251.63**	499.23
*Behavioral symptoms at wave 1*				
Internalizing score (ranges from 0 to 66)	10.63	8.47	9.24***	8.17
Externalizing score (ranges from 0 to 68)	15.89	11.45	13.69***	11.25
*NSCAW I*	78.7%	–	68.0%***	–
Average # of waves aged 12-17 (ranges from 1 to 3)	1.43	0.59	1.71***	0.64
*NSCAW II*	21.3%	–	32.0%***	–
Average # of waves aged 12-17 (Ranges from 1 to 2)	1.25	0.43	1.45***	0.50
*Adolescent outcome measures (Standardized Score)*				
School engagement summary score^a^	− 0.15	1.03	0.11***	0.97
Woodcock-Johnson letter-word identification score^b^	− 0.15	1.06	0.13***	0.93
Woodcock-Johnson applied problems score^b^	− 0.14	1.06	0.12***	0.93
Future expectations summary score^c^	− 0.14	1.10	0.11***	0.91
*N*	2,002 Individuals			

Data is from baseline wave. Estimates are unweighted.

^*^ (**) [***] indicates difference in means is statistically signification at .10(.05)[.01] level. Standard deviation is not calculated for dichotomous and categorical covariates (indicated by “-”). School engagement submeasures are rescaled to always be in a positive direction.

^a^ All 11 items are included: enjoys being in school, hates being in school, tries to do their best, finds work too hard, finds class interesting, fails to complete or turn in work, sent to office or kept after for misbehavior, gets along with teachers, listens carefully, gets homework done, and gets along with other students.

^b^ Denotes the smaller sample size due to first introduction occurring in wave 5 of NSCAW I: 1,738 individuals.

^c^ Items included are based on Exploratory Factor Analysis results (see Appendix A): chances to live to 35, chances to graduate high school, and chances to have a good job by 30.

### Unadjusted Differences in Academic Well-Being

When assessing adolescent academic well-being outcomes, youth with preteen SI tended to fare worse than those without preteen SI, as expected. Specifically, youth who experienced SI at ages 7–11 reported lower school expectations (Cohen’s *d* = − 0.27 [95% CI: − 0.37 – − 0.16]), lower reading and math skills (Letter-Word Identification: Cohen’s *d* = − 0.28 [95% CI: − 0.41 – − 0.14]; Applied Problems: Cohen’s *d* = − 0.26 [95% CI: − 0.39 – − 0.13]), and lower future expectations (Cohen’s *d* = − 0.25 [95% CI: − 0.35 – − 0.15]) in adolescence.

### Adjusted Models Testing Differences in Academic Well-Being

Table 2 shows results from the regression models for the academic well-being measures. Each column represents a different regression model, where the outcome consists of one of the categories of academic well-being: school engagement, academic achievement, and future engagement. For each outcome of academic well-being, a statistically significant negative association was found with preteen SI after controlling for other observed covariates. More specifically, compared to youth without preteen SI, those with preteen SI reported lower School Engagement (Cohen’s *d* = − 0.21 [95% CI: − 0.34 – − 0.09]), lower Letter-Word Identification scores (Cohen’s *d* = −0.22 standard deviations (95% CI: − 0.35 – − 0.09), lower Applied Problems scores (Cohen’s *d* = − 0.17 [95% CI: − 0.31 – − 0.03]), and lower Future Expectations (Cohen’s *d* = − 0.28 [95% CI: − 0.40 – − 0.16]). We found no statistically significant differences based on race/ethnicity or sex.

**Table 2.** Predictors of Adolescent Academic Well Being

	School engagement summary measure	Letter-word identification score	Applied problems score	Future expectations summary measure
	Coefficient estimate (95% confidence interval)			
Suicidal ideation in preteen years	− 0.21*** (− 0.34 – -0.09)	− 0.22*** (− 0.35 – − 0.09)	-0.17** (-0.31 – -0.03)	-0.28*** (-0.40 – -0.16)
Age	− 0.02 (− 0.07 – 0.04)	− 0.05* (− 0.11 – 0.01)	− 0.09*** (− 0.15 – − 0.02)	− 0.05* (− 0.11 – − 0.01)
Male	− 0.50 (− 1.32 – 0.32)	− 0.18 (− 0.95 – 0.51)	− 0.41 (− 1.26 – 0.44)	− 0.36 (− 1.12 – 0.40)
Age X male interaction	0.03 (− 0.06 – 0.11)	0.001 (− 0.08 – 0.08)	0.05 (− 0.04 – 0.14)	0.03 (− 0.05 – 0.10)
*Race/Ethnicity*				
Black, non-hispanic (Ref.)	–	–	–	–
Hispanic	− 0.10 (− 0.26 – 0.05)	0.18** (0.02 – 0.33)	0.05 (− 0.12 – 0.22)	− 0.14* (− 0.30 – 0.01)
Other/multiple, non-hispanic	− 0.03 (− 0.24 – 0.19)	0.29*** (0.07 – 0.51)	0.18 (− 0.06 – 0.42)	− 0.11 (− 0.34 – 0.13)
White, non-hispanic	0.06 (− 0.06 – 0.18)	0.26*** (0.13 – 0.39)	0.20*** (0.06 – 0.33)	− 0.05 (− 0.18 – 0.08)
*Form of abuse*				
Failure to provide (Ref.)	–	–	–	–
Failure to supervise	0.07 (− 0.11 – 0.25)	0.06 (− 0.14 – 0.25)	0.07 (− 0.14 – 0.28)	− 0.01 (− 0.19 – 0.17)
Other	0.13 (− 0.05 – 0.32)	0.13 (− 0.06 – 0.32)	0.19* (− 0.01 – 0.38)	0.08 (− 0.11 – 0.26)
Physical abuse	0.04 (− 0.16 – 0.23)	0.11 (− 0.08 – 0.31)	0.11 (− 0.10 – 0.32)	0.03 (− 0.16 – 0.22)
Sexual abuse	0.11 (− 0.10 – 0.31)	0.03 (− 0.20 – 0.25)	0.08 (− 0.16 – 0.31)	0.03 (− 0.18 – 0.23)
Harm from prior maltreatment	− 0.02 (− 0.07 – 0.03)	0.01 (− 0.04 – 0.07)	− 0.01 (− 0.08 – 0.05)	− 0.01 (− 0.06 – 0.05)
Days in out-of-home placement	0.0001* (− 0.0001 – 0.0002)	0.0001 (− 0.0001 – 0.0002)	− 0.00001 (− 0.0002 – 0.0001)	− 0.000002 (− 0.0001 – 0.0001)
Wave 1 internalizing score	0.01 (− 0.01 – 0.02)	0.01 (− 0.002 – 0.02)	− 0.002 (− 0.01 – 0.01)	0.001 (− 0.01 – 0.01)
Wave 1 externalizing score	− 0.02*** (− 0.03 – − 0.01)	− 0.01*** (− 0.02 – − 0.01)	− 0.01*** (− 0.02 – − 0.002)	− 0.01*** (− 0.02 – − 0.004)
NSCAW-II dummy	0.16*** (0.05 – 0.28)	0.20*** (0.07 – 0.33)	0.30*** (0.16 – 0.43)	0.02 (− 0.10 – 0.14)
*N*	2,002 Individuals (3,048 observations)	1,738 Individuals (1,950 observations)	1,738 Individuals (1,950 observations)	2,002 Individuals (3,048 observations)

^*^(**)[***] indicates estimate is statistically significant at the .10(.05)[.01] level. (Ref.) indicates reference category for a categorical covariate.

Each column represents a separate regression model with a different outcome measure corresponding to academic well-being. The first row of each cell represents the coefficient estimate, while the second row displays the 95% Confidence Interval (CI).

Mixed Effect models accounting for within child clustering and complex sample design. Outcome measures are taken from when children are in adolescence (12–17), while covariate information is gathered from the lookback period (7–11).

## Discussion

Preteen SI in child welfare system (CWS)-involved youth was associated with lower levels of academic well-being in adolescence. Specifically, youth reporting SI between ages 7–11 reported lower levels of school engagement, performed worse on several tests of reading and math skills, and had a less positive outlook for their future achievements as adolescents.

This is the first study, to our knowledge, to examine adolescent academic outcomes of CWS-involved youth who experience preteen SI. This differs from existing literature documenting negative associations between SI and aspects of academic well-being in several respects (Carter et al., 2007; Chen et al., 2022; De Luca & Wyman, 2012; De Luca et al., 2016; Marraccini & Brier, 2017; Posick and Zimmerman, 2022; Zhu et al., 2019). First, prior work in this area exclusively focused on SI occurring in adolescence instead of preteen years. Second, most of these analyses examined how measures of academic well-being predicted SI, whereas this study flips the order of the relationship between the two, using a longitudinal study design to avoid concerns related to reverse causality. We did identify one study which structured their analyses similarly by conceptualizing SI as a predictor of academic achievement, but its focus was on college students (De Luca et al., 2016). Similar to our findings, it observed SI was associated with moderately lower Grade Point Average.

These findings are concerning for many reasons. First, a person’s ability to succeed academically can be critical to their future health, development, and well-being. Prior research has established a bidirectional relationship between school performance and mental health (Esch et al., 2014; von Simson et al., 2022). Our results suggest that in addition to their mental health needs, preteens with SI may benefit from additional academic and school support in order to ensure they can thrive later in life. Lower educational attainment has been found to be associated with lower school engagement and poorer cognitive performance (Abbott-Chapman et al., 2014; Fraysier et al., 2020). These, in turn, contribute to a range of other adverse health outcomes such as earlier mortality (Halpern-Manners et al., 2020; Montez & Hayward, 2014) and chronic disease (Choi et al., 2011; Vable et al., 2019), poorer self-rated health (Schellekens & Ziv, 2020), and greater engagement in risky health behaviors (Pampel et al., 2010). Therefore, offering additional support to preteens with SI, alongside mental health interventions, could ultimately prevent a host of serious long-term consequences.

Youth expectations for the future were significantly lower among those who experienced SI in their preteen years relative to their CWS-involved peers without preteen SI. This too has implications for youth’s present and future health. Several studies show higher expectations for the future among adolescents is associated with a lower likelihood of risky sex (Sipsma et al., 2015), substance use (Prince et al., 2019), and violent behavior (Brumley et al., 2017), as well as higher levels of physical activity (McDade et al., 2011). Other work has shown long-term associations with better physical and mental health in adulthood (Kim & Kim, 2020). Altogether, these mechanisms of poorer school performance and lower optimism for the future reinforce the notion that preteen youth SI should be acknowledged as a serious public health issue, as SI can have lasting effects both physically and emotionally beyond more immediate concerns of increased risk of self-harm and suicide.

### Implications for School Health Policy, Practice, and Equity

These findings also have important implications for mental health service providers and policymakers. They further reinforce the urgency with which children with SI should be identified early and offered needed services and support, including monitoring and intervention for academic in addition to mental health needs. Future studies are needed to examine the mechanisms explaining the association between preteen SI and later academic outcomes; for example, whether improving preteens’ feelings of connectedness to and engagement with school, or receipt of evidence-based suicide prevention/interventions may improve their academic well-being.

While specific interventions/policies were not the focus for this study, other work suggests complementary strategies may mitigate the effects of SI among CWS-involved children. While evidence-based school-based suicide prevention programs can help adolescents (Singer et al., 2019), the field currently lacks evidence for how schools can address SI in younger, elementary aged youth. In one recent article, Ayer and colleagues (2023) identified several school-based programs not originally designed to target suicide risk but which could have potent “crossover effects” or unintended benefits for preventing suicide (Ayer et al., 2023). Many of these could simultaneously promote mental health and academic well-being throughout the K-12 period. For example, fostering a sense of connection within communities can promote more hopeful future expectations among youth (Stoddard & Pierce, 2015) and may also be a protective factor for youth suicide (Whitlock et al., 2014). Building these communal bonds may therefore be especially beneficial to preteens experiencing SI. Consequently, programs that help students feel a sense of belonging and connection with their peers may hold promise and should be investigated further. However, it is important to note the unique challenges in promoting connection and belonging for CWS-involved children who may be transitioning between multiple caregivers, schools, and neighborhoods (Miller et al., 2013). Sources of consistent, strong support in school—particularly emotional and social support (Armstrong et al., 2005; Chu et al., 2010)—may help to mitigate the risks associated with these transitions including suicide risk and other adverse outcomes (Miller et al., 2013). Again, future research is needed to determine best practices for CWS-involved youth that have experienced preteen SI to help avoid poor academic achievement in adolescence and increase hope for future success.

## Limitations

This study has several limitations to acknowledge. First, despite the study’s longitudinal design, the findings described are associations, rather than causal. This is because unmeasured confounders not included in the regression model, such as gender identity, household income, educational attainment, or access to mental health or educational resources, may help explain the true relationship between SI and academic well-being. Notably, measures of academic well-being during the baseline period were not collected throughout ages 7–11 by the data source. Future work could consider implementing an instrumental variable approach to determine presence of a causal effect, though it may prove difficult to identify a variable that is both completely unrelated with academic well-being *and* strongly correlated with SI in early childhood. Second, the item for SI is a single-item measure. Although measuring SI with a single item is common in the literature, this form of instrumentation is more prone to measurement error than those with more precise language, additional items, and multiple response options (Millner et al., 2015). Third, because participants were not followed throughout the entire preteen period, there is an additional form of measurement error that could impact the SI measure. However, we would expect this particular issue to bias our results towards a null finding, since any subsample of children misclassified as not ideating during preteen years would ostensibly shrink the observed differences in the outcomes across the two groups. Fourth, the results for the Future Expectations summary measure should be considered in the context that this outcome was based on an Exploratory Factor Analysis, the results of which can capitalize on chance and are sample specific. Relatedly, despite its usage as a single composite scale, the measure for School Engagement has not been validated in external samples, and there is some debate about whether it is more appropriate to operationalize as several subscales instead (Cage et al., 2019; Cage et al., 2020). Fifth, we note that while use of our analytic weights allowed for us to have nationally representative estimates for the CWS child population in our model, NSCAW I and II both used oversampling of infants, sexual abuse cases, children in out-of-home placements, and cases receiving services that could also lead to overfitting challenges. However, when comparing unweighted and analytic weighted models, we found the direction and magnitude of inferences was maintained. Lastly, as this is a study of CWS-involved youth, the findings may not be generalizable to the broader population of children. Additionally, the NSCAW data were collected decades ago and may not generalize to children growing up in the 2020s. Of particular note, recent developments such as the widespread adoption of smartphone technology and growing importance of social media in young peoples’ lives could have impacted both the prevalence of suicidal ideation among preteens, as well as altered the potential relationship between suicidal ideation and subsequent academic well-being (Anderson et al., 2023; Twenge, 2020).

## Conclusions

There is growing concern that youth in the United States are in the middle of a mental health crisis, including higher levels of suicidal ideation and other suicide-related behaviors (Bridge et al., 2023; Centers for Disease Control and Prevention, 2024; Office of the Surgeon General, 2021). However, suicide risk among preteens, as well as its longer-term impact on health and flourishing, has been poorly understood, particularly within high-risk populations. Using a nationally representative longitudinal study of youth involved in the U.S. child welfare system, we find evidence that SI experienced in preteen years is associated with poorer academic well-being in adolescence, including lower levels of school engagement, poorer achievement in reading and mathematics, and reduced expectations for one’s future prospects in life. These findings build upon previous research articulating the importance of identifying and addressing suicidal thoughts early in life to prevent suicide and other poor mental health outcomes, by demonstrating that the potential adverse impacts may be felt more broadly across the lifecourse.

## Appendix A. Exploratory factor analysis findings

The School Engagement and Future Expectations outcomes consist of a number of items (11 and 5 respectively). Rather than include all the items as a summary measure *a priori*, we conduct an exploratory factor analysis (EFA) to assess whether a subset of the items might serve as a more appropriate indicator of the latent construct being measured. This is especially salient for future expectations items, which have a within-sample Cronbach’s alpha of α=0.66 (the corresponding statistics for the School Engagement items is α=0.80, suggesting that a summary measure using all items is reasonably appropriate).

Since participants may report multiple outcomes during the study, we restrict the EFA procedure to the first observation at which the youth is aged 12–17. Over the 100 imputations developed to address missingness in the data, we run a factor analysis using a polychoric correlation matrix to account for the ordinal nature of the response categories. Eigenvalues from unrestricted analyses suggest that a two-factor solution was appropriate for school expectations items and a one-factor solution for Future Expectations measures. Admittedly, the second factor for school expectations falls below the conventional eigenvalue of 1, but we opt to include it since there is a notable dropoff from its eigenvalue (0.63) to that of the next factor (0.22). We do not do this for future expectations, since the second potential factor in that solution is only comprised of two items.

Preliminary results – future expectations measures See Tables

**Table 3:** Polychoric correlation structure from first imputation

	Live to 35	Married at 25	Graduate high school	Good Job by 30	Have a child and raise family
Live to 35	–				
Married at 25	0.1554	–			
Graduate high school	0.4159	0.1757	–		
Good job by 30	0.3868	0.1895	0.5788	–	
Have a child and raise family	0.2717	0.4014	0.2666	0.3359	–

3 and

**Table 4:** Eigenvalues from unrotated exploratory factor analysis from first imputation

	Eigenvalues
Factor 1	1.52
Factor 2	0.29
Factor 3	− 0.07
Factor 4	− 0.18
Factor 5	− 0.24

^4^*.*

Preliminary results – school engagement measures See Tables

**Table 5:** Polychoric correlation structure from first imputation

	Enjoy school	Hate school	Try best	Too hard	Find interesting	Don’t turn in work	Sent to office	Get along w/ teachers	Listen carefully	Get homework done	Get along w/ peers
Enjoy school	–										
Hate school	0.5901	–									
Try best	0.2608	0.2056	–								
Too hard	0.1226	0.1750	0.1814	–							
Find interesting	0.4651	0.3850	0.2438	0.0810	–						
Don’t turn in work	0.0819	0.1854	0.2309	0.1695	0.0957	–					
Sent to office	0.2202	0.2530	0.2797	0.0973	0.2117	0.2430	–				
Get along w/ teachers	0.4031	0.3509	0.3529	0.1285	0.3531	0.1347	0.4265	–			
Listen carefully	0.3244	0.2949	0.5365	0.1252	0.3245	0.1790	0.3450	0.4371	–		
Get homework done	0.2590	0.2338	0.4592	0.1476	0.2933	0.3283	0.3298	0.3353	0.4710	–	
Get along w/ peers	0.2203	0.2196	0.1690	0.1477	0.2629	0.0469	0.2050	0.3052	0.2298	0.2264	–

5 and

**Table 6:** Eigenvalues from unrotated exploratory factor analysis from first imputation

	Eigenvalues
Factor 1	3.10
Factor 2	0.63
Factor 3	0.22
Factor 4	0.15
Factor 5	0.06
Factor 6	0.01
Factor 7	− 0.12
Factor 8	− 0.14
Factor 9	− 0.18
Factor 10	− 0.19
Factor 11	− 0.23

^6^.

After rerunning the factor analysis with these restrictions, we rotated the factor solution using the *oblimin* algorithm, which allows for factors to be correlated with one another. The average estimates across the 100 imputations is presented below. For inclusion in the summary measure, we adopt a cutoff threshold of 0.3 (bolded in Tables

**Table 7:** Rotated factor loadings – future expectations measures

	Factor 1	Factor 2 (not used)
Live to 35	**0.4639**	0.1041
Married at 25	− 0.0548	0.5297
Graduate high school	**0.7277**	− 0.0630
Good job by 30	**0.6564**	0.0674
Have a child and raise family	0.0835	0.5323

For inclusion in the summary measure, we adopt a cutoff threshold of 0.3 are given in bold

7 and

**Table 8:** Rotated factor loadings – school engagement measures

	Factor 1	Factor 2 (not used)
Enjoy school	− 0.0263	**0.7495**
Hate school	− 0.0031	**0.6764**
Try best	**0.6702**	− 0.0366
Too hard	0.1724	0.1222
Find interesting	0.0820	**0.5323**
Don’t turn in work	**0.4492**	− 0.1033
Sent to office	**0.4756**	0.1055
Get along w/ teachers	**0.3610**	0.3439*
Listen carefully	**0.6483**	0.0885
Get homework done	**0.7010**	− 0.0385
Get along w/ peers	0.1415	0.2778

For inclusion in the summary measure, we adopt a cutoff threshold of 0.3 are given in bold

^*^ = Don’t include in this factor since it is included in the first factor

8).

Results from this procedure suggest a 3-item summary measure serves well for Future Expectations (chances to live to 35, chances to graduate high school, and chances to have a good job). Although the Cronbach’s Alpha for the school engagement items suggests a summary measure using all 11 items is reasonable, results from the EFA suggests two subscales, which we additionally analyze (Academic Behaviors Subscale: try their best work in school, fail to complete or turn in assignments, are sent to the office or stay after school due to misbehavior, get along with their teachers, listen carefully or pay attention, get their homework done; Academic Happiness Subscale: enjoy being in school, hate being in school, and find class interesting).
